# Current Knowledge on CRISPR Strategies Against Antimicrobial-Resistant Bacteria

**DOI:** 10.3390/antibiotics13121141

**Published:** 2024-11-27

**Authors:** Carlos de la Fuente Tagarro, Diego Martín-González, Andrea De Lucas, Sergio Bordel, Fernando Santos-Beneit

**Affiliations:** 1 Department of Chemical Engineering and Environmental Technology, School of Industrial Engineering, University of Valladolid, Paseo Prado de la Magdalena 3-5, 47011 Valladolid, Spain; carlos.fuente.tagarro@uva.es (C.d.l.F.T.); diego.marting@uva.es (D.M.-G.); andrea.lucas@uva.es (A.D.L.); sergio.bordel@uva.es (S.B.); 2 Institute of Sustainable Processes, Paseo Prado de la Magdalena 3-5, 47011 Valladolid, Spain

**Keywords:** antibiotic, AMR, multidrug-resistant bacteria, CRISPR/Cas, phages, nanoparticles, *Streptomyces*, BGCs

## Abstract

CRISPR/Cas systems have emerged as valuable tools to approach the problem of antimicrobial resistance by either sensitizing or lysing resistant bacteria or by aiding in antibiotic development, with successful applications across diverse organisms, including bacteria and fungi. CRISPR/Cas systems can target plasmids or the bacterial chromosome of AMR-bacteria, and it is especially necessary to have an efficient entry into the target cells, which can be achieved through nanoparticles or bacteriophages. Regarding antibiotic development and production, though the use of CRISPR/Cas in this field is still modest, there is an untapped reservoir of bacterial and fungal natural products, with over 95% yet to be characterized. In *Streptomyces*, a key antibiotic-producing bacterial genus, CRISPR/Cas has been successfully used to activate silent biosynthetic gene clusters, leading to the discovery of new antibiotics. CRISPR/Cas is also applicable to non-model bacteria and different species of fungi, making it a versatile tool for natural products discovery. Moreover, CRISPR/Cas-based studies offer insights into metabolic regulation and biosynthetic pathways in both bacteria and fungi, highlighting its utility in understanding genetic regulation and improving industrial strains. In this work, we review ongoing innovations on ways to treat antimicrobial resistances and on antibiotic discovery using CRISPR/Cas platforms, highlighting the role of bacteria and fungi in these processes.

## 1. Introduction

### 1.1. The Issue of Antibiotic Resistance

Since the discovery of the first antimicrobial compound, the use of these therapeutic drugs has increased steadily [1]. The widespread use, and in most cases misuse, of antimicrobial drugs has led to a global problem: the rise of antimicrobial resistance (AMR) [2]. AMR can be defined as the insensitivity of microorganisms to an antimicrobial compound to which they were previously vulnerable [3]. Over time, microbial infections caused by multidrug-resistant microorganisms have increased, while antimicrobial treatments have lost their effectiveness [4]. This problem especially compromises those with chronic complications or those receiving immunosuppressive treatment, who largely depend on antimicrobials to combat infections.

Among antimicrobial-resistant microorganisms, bacteria are of special importance. The most recent global assessments estimate that, in 2019, almost 5 million deaths were associated with bacterial AMR, close to 1.3 million of which could have been prevented worldwide if the infecting bacteria had not been resistant to antimicrobials [5]. The World Health Organization (WHO) publishes the Bacterial Priority Pathogens List to identify the most threatening antibiotic-resistant bacteria and to direct research towards a solution [6]. These bacteria include methicillin-resistant *Staphylococcus aureus* (MRSA), associated with high mortality rates each year worldwide, especially in nosocomial infections (infections that take place in a healthcare facility) [7], and multidrug-resistant Gram-negative bacteria (MDR-GNB) [8].

In this context, the development and improvement of therapeutic compounds to combat bacterial infections represents a priority for healthcare professionals and the pharmaceutical industry. A great part of the effort in pharmaceutical investigations to discover new antibiotics with which to confront this threat is focused on microbial resources [9], since they have been a reliable source of new antibiotics, alongside other natural products (NPs), for a long time [10].

### 1.2. Relationship Between CRISPR/Cas and AMR in Bacteria

The presence of resistance genes in multidrug-resistant bacteria may be due to mutations or to the acquisition of exogenous DNA in the form of mobile genetic elements (MGEs). MGEs can remain as independent structures from the genome, being able to conjugate in some cases (horizontal transmission), or they can be integrated into the genome thanks to integrative conjugative elements (resulting in vertical transmission as the bacteria grows). Some human pathogens are naturally competent, like *Streptococcus pneumoniae*, *Helicobacter pylori*, *Acinetobacter baumannii*, *Vibrio cholerae*, and *Neisseria gonorrhoeae* [11,12], while others can acquire MGEs through conjugation or transduction. MGEs normally contain different types of antibiotic resistance genes, as well as other genetic structures, such as integrons, that allow the addition or exchange of resistance genes in the MGEs [13,14]. Due to the fact that exogenous DNA can be harmful to the host bacteria, as it may be viral genetic material, around 40% of bacteria present a CRISPR/Cas system as a defense mechanism [15].

CRISPR/Cas systems consist of DNA sequences that are found on archaea and bacteria as a part of their adaptive immunity [16]. A CRISPR locus is composed of a CRISPR array located in between various *cas* genes. The array is made up of short repeats, separated by short unique DNA sequences known as spacers [17]. CRISPR/Cas immunity works in three sequential steps, adaptation, expression/maturation, and interference [18,19], all of which require specific Cas proteins encoded near the CRISPR array as well as other auxiliary proteins [20]. Essentially, fragments of foreign DNA are incorporated as spacers to the array, and later expressed as RNAs that guide Cas proteins to cleave extrinsic nucleic acids when they enter the bacteria [21]. The CRISPR/Cas systems are arranged into six types based on physiology and modes of operation, with two primary classifications, Class 1 and Class 2 [17]. Class 1 systems include types I, III, and IV, which use Cas protein complexes for the identification and cleavage of nucleic acids. Class 2 systems, which include types II, V, and VI, rely on only one effector protein that carries out both target identification and nucleic acid-cutting tasks [20].

Given this defense mechanism against exogenous DNA, it was first thought that CRISPR/Cas systems could interfere with the acquisition of MGEs, and therefore with the spreading of AMR in bacteria populations and the appearance of multidrug-resistant bacteria.

This idea was supported by research on various bacteria. For example, *Escherichia coli* isolates with the Type I-F CRISPR/Cas system, one of the two types present in *E. coli*, were more susceptible to antibiotics [22]. In *Klebsiella pneumoniae*, clinically relevant strains do not have CRISPR/Cas systems, are competent, and contain more plasmids with antibiotic resistances [23,24,25]. In another study with *Enterococcus faecalis*, the same conclusion was reached. Strains with CRISPR/Cas systems were sensitive to a wide variety of antibiotics such as ampicillin, vancomycin, and erythromycin, among others [26], as the *cas9* gene loss in most antibiotic-resistant *E. faecalis* isolates is a determinant for the presence of conjugative plasmids with resistance genes [27]. In addition, *Acinetobacter baumannii* strains that did not present an active CRISPR/Cas system had more antibiotic resistance genes [28,29].

However, not all studies report this negative correlation between the presence of CRISPR/Cas systems and antibiotic resistance. A study on *E. coli* isolates found that, in general, CRISPR/Cas systems had no effect on the presence of MGEs with antibiotic resistance [30], and in the *Enterococcus* genus, only one of the ten resistance genes analyzed was more prevalent in isolates without a CRISPR/Cas system [31]. And when analyzing CRISPR/Cas and antibiotic resistance genes in more than five thousand bacteria, there was no association in most of them, with only clinically relevant species presenting a correlation [32], consistent with a more recent analysis [33].

In addition to the CRISPR/Cas activity on exogenous DNA, CRISPR/Cas systems can influence antibiotic resistance, and bacterial survival in general, through the regulation of other processes. Shabbir et al. studied the CRISPR/Cas9 system in *Campylobacter jejuni* NCTC11168 and concluded that the *cas9* gene was involved in regulating the ribosomal proteins, promoting antibiotic resistance, as well as in expressing membrane proteins [34]. Regarding the same gene and the bacterial envelope, in the case of *Francisella novicida* CRISPR/Cas9 regulates the synthesis of a membrane lipoprotein as a response to damage caused by antibiotics that act on the cell wall, improving envelope integrity [35]. And in the aforementioned *A. baumannii*, CRISPR/Cas, through the degradation of the mRNA of quorum sensing regulator *abaI*, inhibits the expression of multidrug efflux pumps and the formation of biofilms, which grant resistance to antibiotics [29].

The fact that CRISPR/Cas systems produce such dissimilar results is due to several facts. One of them is that although these systems protect against exogenous DNA, when a strong stress caused by the presence of antibiotics arises CRISPR/Cas action may be altered or inactivated, allowing the entrance of DNA from the environment [36]. In other cases, there may be mutations in *cas* gene clusters that affect their function [36]. This is the case in *Staphylococcus epidermidis*, which contains a specific CRISPR/Cas system, called type III-A, that produces mutations on the genome due to non-specific DNase activity [37]. This way, microorganisms with a type III-A CRISPR/Cas system may develop AMR without depending on acquiring exogenous resistance genes. Anti-CRISPR proteins have been evolved by bacteriophages to avoid CRISPR immunity [4], and can therefore indirectly affect the spread of AMR-conferring plasmids by interfering with CRISPR. These anti-CRISPR proteins can also be found in other mobile genetic elements like plasmids, so they can themselves prevail after entering a bacteria cell [38]. What seems clear is that this variability in CRISPR/Cas effects is due to multiple factors such as bacterial species, types of antibiotics, host (in the case of pathogenic bacteria), or strain isolation time, among others [36]. Even the environment is influential, as the antiplasmid activity of CRISPR/Cas in *E. faecalis* is absent in vitro [39], adding on to the complexity of these systems and displaying the limitations of in vitro models when studying pathogenic bacteria.

### 1.3. CRISPR/Cas: A Genetic Tool of Increasing Interest

The use of the CRISPR/Cas system in the pharmaceutical industry is becoming one of the most cutting-edge strategies [40], and its applications in the topic of antibiotics are also increasingly relevant. It can be used both as a genetic tool for the development and production of microbial NPs with antimicrobial properties and as a therapeutic agent by modifying multidrug-resistant microorganisms to make them vulnerable. In the case of NPs, sequencing technologies and computational resources are able to identify numerous NP biosynthetic gene clusters (BGCs) from the genomes of different bacteria. However, most of these BGCs are silent or poorly expressed in native strains and remain to be activated and investigated.

**Figure 1:**
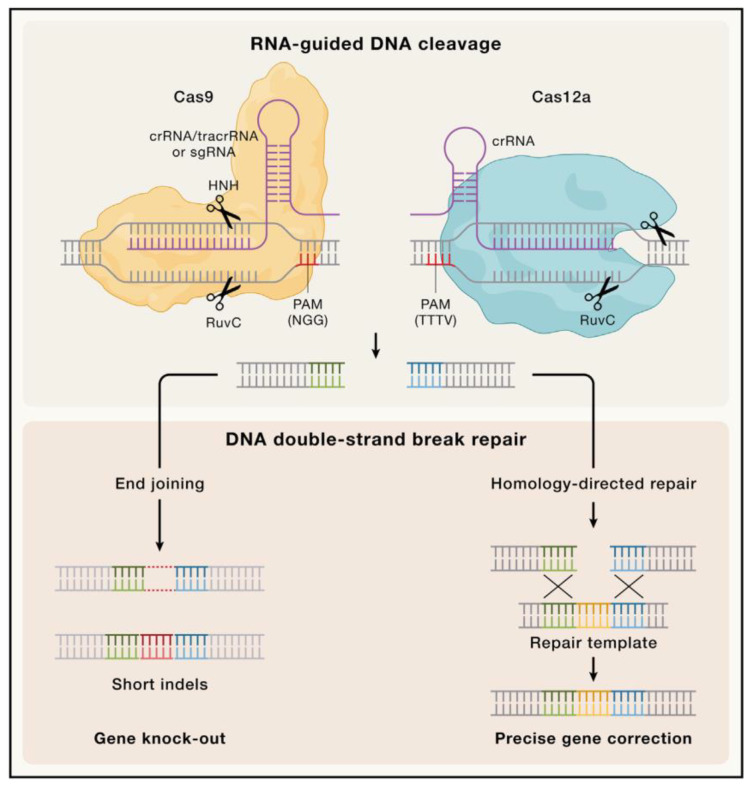
Molecular principles of CRISPR genome editing. CRISPR genome editing relies on RNA-guided nucleases such as Cas9 and Cas12a for site-specific target DNA recognition and cleavage. Cas9 utilizes a dual-guide RNA composed of a CRISPR RNA (crRNA)-*trans*-activating CRISPR RNA (tracrRNA) pair or a single-guide RNA (sgRNA), whereas Cas12a is programmed with a crRNA only. Target DNA recognition is dependent on complementarity with the spacer sequence of the guide RNA as well as the presence of a protospacer adjacent motif (PAM). Cas9 recognizes an NGG PAM, whereas Cas12a requires a TTTV PAM (V = G, C, or A). Upon target binding, the nucleases catalyze DNA cleavage, generating a DNA double-strand break (DSB). DSB repair by cellular DNA repair pathways leads to the introduction of genetic modifications (edits). The end-joining pathways result in short insertions or deletions (indels), whereas homology-directed repair (HDR) using an exogenous DNA repair template can be used to engineer precise modifications. The green and blue colours in the figure show homology with a repair template, and the red and yellow colours represent a random sequence and a template sequence, respectively. Reproduced from [42] under a CC BY 4.0 license.

This natural phenomenon was repurposed into a genetic engineering tool for research and clinical applications. Its usefulness is based on efficient directed genome editing in cells thanks to a specific sequence of few nucleotides within a guide RNA [41]. CRISPR/Cas 9 (type II) and Cas12 (type V) are the most used CRISPR systems in genetic engineering, and their mechanisms are illustrated in Figure 1. Briefly, Cas9 requires either a dual-guide RNA, made of a CRISPR RNA (crRNA) and a *trans*-activating CRISPR RNA (tracrRNA), or a single-guide RNA (sgRNA), while Cas12a only needs a single crRNA. In either case, the spacer located in the guide allows the Cas protein to recognize a specific complementary sequence and cleave it, resulting in a double-strand break. As the bacterial repair systems activate at this point, the addition of an exogenous template allows for the insertion of designed sequences [42].

In addition to CRISPR/Cas, there are other previously discovered gene editing and regulation tools, such as transcription activator-like effector nucleases (TALENs) and zinc-finger nucleases (ZFNs). Both produce double strand breaks (DSB) at specific locations in the genome which initiate DNA repair by the cellular machinery, allowing for the introduction of specific mutations or genetic modifications [43]. To achieve this, TALENs use a transcription activator-like (TAL) effector and the nuclease domain of the restriction enzyme FokI [44]. ZFNs also have the nuclease domain of FokI, but they recognize specific DNA sequences thanks to a zinc finger protein [45]. Their main disadvantages are the higher chance of mutating off-target sites compared with CRISPR technology and the need to produce new proteins for each new DNA target. In contrast, CRISPR/Cas systems maintain the same protein (Cas9, Cas12, etc.) and are able to modify multiple targets at the same time because different sgRNA can be introduced simultaneously [20,46]. For these reasons, CRISPR/Cas systems have a remarkably higher accuracy and efficiency for genome editing of various model organisms than other strategies, making them a powerful tool for the discovery, characterization, reengineering, and production of potential pharmaceutical drugs [47].

**Figure 2:**
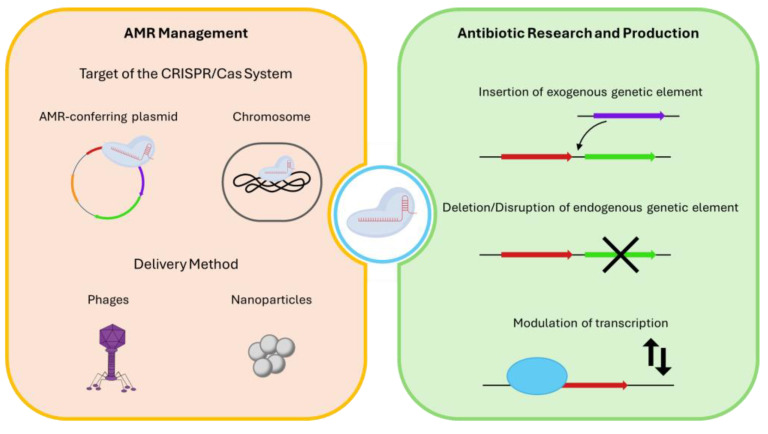
Overview of the two CRISPR/Cas strategies to combat antibiotic-resistant bacteria: AMR management and antibiotic research and production.

Considering the advantages of CRISPR/Cas systems when compared to other genetic engineering strategies, and existing literature on their application in the subject of antibiotic resistance, this review aims to examine the use of CRISPR/Cas strategies in AMR management, with a special focus on antibiotic research and production. A schematic overview of the use of CRISPR/Cas strategies to manage AMR and develop antibiotics is presented in Figure 2.

New research tends to focus more on the elimination of antibiotic resistance or the search for alternative therapeutic agents [48,49], but antibiotic development is still a necessity, and CRISPR/Cas tools can be a powerful tool to better understand how organisms synthesize antibiotics and how we can improve their production and discover new ones.

## 2. CRISPR/Cas Systems Applied to AMR Management

Literature on the topic of AMR management with CRISPR/Cas systems is extensive and focuses mostly on sensitizing bacteria to antibiotics. The elements of the CRISPR/Cas systems can vary, but these approaches share the need for a target gene, which may be located on an AMR resistance-conferring plasmid or on the bacteria’s genome, and for an efficient delivery method, which can be achieved with bacteriophages or nanoparticles (Figure 2).

### 2.1. CRISPR/Cas Without Delivery Vehicles

The works mentioned below involve introducing CRISPR/Cas systems to competent strains in vector form. Some studies directly eliminate the resistance genes in bacteria, cleaving them with CRISPR/Cas machinery. Tao et al. [50] eliminated plasmids containing carbapenem resistance in *E. coli* using a CRISPR/Cas9 system targeting the *bla*_KPC−2_ gene. Wu et al. [51] also used a CRISPR/Cas9 system to resensitize to carbapenem, but on *Shewanella algae*, by targeting a *bla*_OXA-55_-like gene. A different strategy is to target a different element of the plasmid that harbors the resistance gene. Sodani et al. [52] determined that CRISPR/Cas9 can resensitize *Mycobacterium smegmatis* to hygromycin resistance by targeting the plasmid containing the resistance gene.

Some approaches involve the use of CRISPR/Cas elements endogenous to the bacteria species. Zhou et al. [53] developed a conjugative plasmid with a CRISPR/Cas3 system native to *Klebsiella pneumoniae*, and studied its ability to eliminate IncFII plasmids—responsible for the dissemination of multiple antibiotic resistance in the species—and prevent infection in vivo. They found that this system is very effective at both eliminating plasmids with resistance genes present in bacteria populations and preventing their dissemination. Benz et al. [54], instead of introducing a complete CRISPR/Cas system, took advantage of a Type IV-A3 CRISPR/Cas system native to *K. pneumoniae* conjugative plasmids. These plasmids are abundant in the species, and their CRISPR/Cas system interferes with the establishment of other conjugative plasmids through transcriptional repression. The researchers designed crRNA to direct the CRISPR/Cas system to specific genes and found that targeting a β-lactam resistance gene in the genome of *K. pneumoniae* successfully resensitized the bacteria. The following case involves introducing only the guiding element into the bacteria, and therefore relying on the CRISPR/Cas proteins already present in the bacteria. Xu et al. [55] introduced plasmids with crRNA guides for multiple resistance genes of *P. aeruginosa*, deleting them and sensitizing the bacteria.

The use of CRISPR/Cas for AMR management is not limited to only the CRISPR/Cas elements. He et al. [56] made use of a transposase responsible for AMR dissemination in Gram-negative bacteria and constructed a CRISPR-containing transposon system. This transposon successfully integrated into the genome of *E. coli*, and the CRISPR/Cas system carried was able to eliminate plasmids granting AMR, as well as to prevent the acquisition of conjugative plasmids.

### 2.2. CRISPR/Cas with Delivery Vehicles

One of the most important factors to consider is the delivery of the CRSIPR/Cas system into the bacteria. This is especially relevant in the case of pathogenic bacteria, as an inefficient delivery would allow bacteria to escape and persist inside the host. In vitro, transformation and conjugation are useful techniques, but for the treatment of bacterial infections there are two main approaches: the use of bacteriophages and the use of nanoparticles.

#### 2.2.1. Bacteriophages

Nath et al. [57] mention the potential design of lytic phages as therapeutic agents against pathogens. They support the idea that a cocktail of non-replicating bacteriophages could be used as vectors which contain a plasmid with a CRISPR cluster. The main advantage of this method is that phages only infect bacteria, therefore being harmless to humans or other animals [58].

Using phages to control bacteria has previously been tested in the laboratory. Recently, in 2022 and 2024, respectively, Shahin et al. [59] and Shin et al. [60], worked with *E. coli* strains that contain a plasmid with resistance to colistin (due to the presence of the *mcr-1* gene). Each team isolated a cocktail of three bacteriophages and lysed the *E. coli*, preventing them from growing in chicken meat and cherry tomatoes. By applying CRISPR/Cas systems, phages may be personalized to control specific bacterial populations [61]. This was carried out by Yosef et al. [62], where CRISPR/Cas9 was used to modify temperate phages. These bacteriophages, containing a CRISPR/Cas cassette, were introduced into different pathogenic *E. coli* strains to eliminate resistance-conferring plasmids, obtaining sensitive strains. The same result was obtained by Sodani et al. [52], who deleted the hygromycin resistance in *Mycobacterium smegmatis* mc2-155. MRSA can also be neutralized by using modified temperate phages [63]. Continuing with temperate phages, Selle et al. [64] obtained a CRISPR/Cas3 recombinant lytic phage from a temperate one to destroy the chromosome of *Clostridioides difficile* at higher efficiency than the *wild-type* phage.

A different approach was used by Qin et al. [65], who used anti-CRISPR-modified bacteriophages against *Pseudomonas aeruginosa* PA14 (with resistance against ampicillin, kanamycin, gentamicin, and streptomycin). Sometimes, to prevent its genetic material from being degraded by CRISPR, phages undergo mutations in the PAM sequence or the spacer or develop anti-CRISPR proteins [66]. Following this idea, phages were modified with anti-CRISPR genes (*acrIF1*, *acrIF2*, and *acrIF3*) and successfully infected *P. aeruginosa* and suppressed antibiotic resistance.

These studies show that the use of CRISPR with phages, while it may be different depending on the strain and the resistance, can adequately sensitize multi-resistant strains or even lyse them and prevent their growth. In some cases, the use of modified phages is suggested to be used on the surface of hospitals, to replace multidrug-resistant strains with those that are not [62]. Some of the main drawbacks of in vivo use of phages are their immunogenicity and the lack of a regulatory framework [67]. Another limitation is that phages can have very narrow host ranges, which could allow some bacteria to escape infection, and therefore treatment, so modifying phages to broaden their host range is of interest [68]. To achieve this, phages can be engineered to bind to more common bacteria receptors, such as lipoproteins in Gram-negative bacteria and peptidoglycan in Gram-positive bacteria [69], or a phage cocktail can be used [70]. Phages can also alter the host microbiome, though the extent to which it may be affected has yet to be determined, so carefully designing phages to avoid detrimental outcomes is essential [71].

#### 2.2.2. Nanoparticles

As the use of phages has its limitations, a non-viral strategy of interest is the use of nanoparticles. Nanotechnology has also proven useful with a wide variety of applications in different fields, like biomedical research and in the food industry against pathogens. There are different types of nanoparticles depending on their size, composition and shape, and some of the better known ones are liposomes, polymers, gold, iron oxide, and albumin [72]. Their sizes vary from 1 nm to 100 nm, and they have a high surface/volume ratio and reactivity [73].

Against pathogens, nanoparticles have proven to act satisfactorily due to five characteristics: (i) they can directly penetrate and damage the cell membrane, (ii) they can carry antibiotics, making their delivery easier, (iii) they are more persistent than antibiotics in the human body, (iv) they produce reactive oxygen species (ROS) and generate oxidative stress, (v) and due to their different nature, they can be changed depending on the objective [74,75]. Kaur et al. used zinc oxide (ZnO) nanoparticles to lyse *E. coli* and *S. aureus* 5021. They concluded that ZnO interacted with the cell membrane, provoking a leakage of cytoplasm. It is thought that this occurred due to the ROS produced by ZnO [76]. Ag and Au nanoparticles have shown to have a similar bacteriostatic action against *E. coli*, *S. aureus* and other food pathogens such as *Listeria monocytogenes*, *Klebsiella pneumoniae*, *Salmonella enterica*, and *Vibrio cholerae* [77,78]. Ag’s action prevents the permeabilization of the cell membrane, while Au’s action causes a reduction in the membrane potential, decreasing ATP production.

Based on these previous studies, CRISPR/Cas has been used in conjunction with nanoparticles to improve their entry into the bacteria. In two studies, sgRNAs and a plasmid with a CRISPR system were protected by liposomes and a polymer to avoid possible degradation before entering the bacteria, and a greater amount of knock-out colonies was obtained when a liposome had been used [79,80]. In another study, Gupta et al. [81] used carbon quantum dots as a carrier to target urinary tract infections. Bacteria adhere to the tissue through fimbrial adhesion virulence factor, corresponding to the *papG* gene. An sgRNA was designed to target this gene and was linked together with a CRISPR system to the carbon nanoparticles, resulting in a reduction in *papG* expression and pathogenicity. In another study, sgRNA and Cas9 protein were introduced into a polymer to target the *mecA* gene of *S. aureus*, responsible for methicillin resistance. The result was that the complex inhibited the growth of the bacteria [82].

Although the introduction of the genetic machinery system is facilitated, nanoparticles have the disadvantage of having to package all the CRISPR/Cas system. Furthermore, when used in vivo, the carrier can produce immunogenicity, although it can be changed to a less immunogenic one, such as lipids [83].

## 3. CRISPR/Cas Systems Applied to Antibiotic Research and Production

Despite the efforts made to develop alternative treatments for AMR bacteria, antibiotics are still essential, and their development is still a necessity. We have found that studies that make use of CRISPR/Cas systems for antibiotic research and production are limited, as CRISPR/Cas technologies are but one of many genetic engineering tools available and their therapeutic use is more attractive. However, they have been used for a variety of purposes, from identifying genes and regulators responsible for antibiotic production to improving yields in industrial strains, and even granting the ability to produce antibiotics to strains that did not originally do so. This is mainly achieved by inserting an exogenous genetic element, knocking out an endogenous genetic element, or even regulating transcription levels (Figure 2).

Among the articles that report the use of CRISPR/Cas systems in bacteria and fungi to—sometimes unintentionally—discover new antibiotics or to modify their production, most of the investigations are performed on bacteria from the *Streptomyces* genus. Actinomycetes, which include *Streptomyces*, stand out because they are natural producers of more than half of the antibiotic classes that are currently used [10]. This is because their long genome allows them to possess the pathways to synthesize antibiotics with different molecular structures [84]. In many cases, these pathways are still to be studied, making them an interesting research subject too. In addition to Actinomycetes, other bacteria and even fungi have also been objects of study. All the microorganisms mentioned in this review are summarized in Table 1.

### 3.1. Research on Bacteria

#### 3.1.1. Streptomyces

Knowing that the *Streptomyces* genus is an important source of antibiotics [97], multiple studies have been performed on them.

Firstly, in the work of Lim et al. [85], gene clusters were activated by CRISPR/Cas9 technology in *Streptomyces roseosporus* NRRL 15 998 to find out if there were other antibiotic BGCs in addition to the already known ones. Previously, it was predicted that *S. roseosporus* NRRL 15998 had more than 20 silenced BGCs. Of all these clusters, one had been found homologous to a type I polyketide synthase cluster from *Streptomyces* sp. ML694-90F3. CRISPR/Cas9 was used to introduce the kasO* promoter, a strong promoter in *Streptomyces* [98]. This modification resulted in the expression of a transcriptional activator, *aurR1*, which in turn activated the expression of the cluster and generated metabolites that were not present in the *wild-type* strain. Through the activation, the authors described and finally isolated auroramycin. It is an antibiotic with antibacterial activity against Gram-positive bacteria similar to that of vancomycin. It has been successfully tested against *Enterococcus faecalis* and MRSA, becoming a potential antibiotic to be used instead of or along with current ones.

Another article about *Streptomyces* focusing on BGCs is the one of Low et al. [86]. Here, they work with *Streptomyces* sp. SD85 to determine the function of one of its 52 biosynthetic gene clusters, BGC11. Through genome sequencing, it was thought that this cluster synthesized sceliphrolactam, a polyene macrolactam, because it supposedly encodes a type I polyketide synthase and other genes, such as regulators and transporters. To corroborate its function, the gene *sceN*, thought to be the synthase, was *knocked-out*. After its deletion, the mutant did not produce the antibiotic, showing that *sceN* and the cluster are directly involved in the synthesis of this antibiotic. Another fact that supports this cluster being responsible for sceliphrolactam production is that some of its genes (*sceG-N*) present a similar homology to *vinH-O* of *Streptomyces halstedii* HC-34, which are involved in the synthesis of vicenistatin, another antibiotic.

The identification of transcriptional regulators and their modification in *Streptomyces* was performed by Liang et al. [87]. They studied the production of actinomycins in *Streptomyces antibioticus* ZS, a *wild-type* strain that naturally produces actinomycins D and V. They identified the gene cluster responsible for actinomycin biosynthesis and its regulator *actO*, used a CRISPR/Cas9 system to make a deletion mutant of *actO*, and observed that the mutant did not produce actinomycins. By overexpressing *actO* in another mutant—through a different technique—and observing a high actinomycin production yield as well as a higher expression of the entire cluster, they confirmed that *actO* acts as a positive regulator of the actinomycin biosynthesis gene cluster.

The last article that we include in the table that mentions the use of *Streptomyces* is the one of Jia et al. [88] with the bacterium *Streptomyces rimosus*. Their goal was to obtain a modified strain with a greater production of oxytetracycline, an aromatic polyketide antibiotic. Modifications were carried out through CRISPR/Cas9, separately or together, in the *zwf2* and *devB* genes, whose products are involved in the oxidative pentose phosphate pathway (PP pathway) and are responsible for hydrolyzing 6-phosphogluconolactone, respectively [99,100]. In previous studies it was determined that *zwf2* was also related with oxytetracycline production [101]. They obtained a double mutant capable of producing 36.8% more oxytetracycline than the *wild-type* strain.

#### 3.1.2. Other Actinomycetes

Other actinomycetes, such as *Saccharopolyspora erythrea* and *Amycolatopsis orientalis*, are also important sources of antibiotics.

On the one hand, *S. erythraea* is a natural producer of erythromycins. These polyketide antibiotics are synthesized by the *ery* gene cluster in the *wild-type* strain NRRL 23338, in low quantities due to low transcription levels of six specific genes. Through a CRISPR/Cas9 system, Zhang et al. replaced the native promoters of those six genes with heterologous promoters of different strengths and studied the production of erythromycins with different promoter combinations. Not only did they see an increase in antibiotic production, but they also gave new insights into the complexity of secondary metabolite biosynthesis, as using the strongest promoters for every gene did not always result in the highest yield [89]. On the other hand, *A. orientalis* is a producer of vancomycin, an antibiotic that has an intriguing regulation by the phosphate concentration of the environment [102] and that shares the same skeleton as the oritavancin precursor A82846B. Qian et al. developed a CRISPR/Cas12a system for the genetic modification of the bacteria and substituted the enzymes responsible for glycosylation in vancomycin biosynthesis with the corresponding ones for A82846B synthesis, resulting in A82846B production and an absence of vancomycin production [90].

#### 3.1.3. Other Classes of Bacteria

Other studies have been performed on bacteria from a different class than Actinomycetes. *Bacillus subtilis* is an antibiotic-producing bacteria with an increasing interest, especially for the production of peptide antibiotics. Among these is fengycin, a lipopeptide initially described as antifungal that also has antibiotic activity [103]. This antimicrobial is produced by the *pps* operon, but yields are very low. Yin et al. [91], after restoring the natural ability of *B. subtilis* 168 to produce fengycin, substituted the weak promoter of the *pps* operon with the strong P*_veg_* promoter with a CRISPR/Cas9 system, and observed an increased production of fengycin.

Using both *Streptoymces species* and *Bacillus subtilis*, Enghiad et al. [95] developed a cloning platform with the aim of discovering new antimicrobial compounds. Unlike other studies, which made use of Cas9, Cas12 was used. Their platform, named CAPTURE (Cas12a-assisted precise targeted cloning using in vivo Cre-*lox* recombination), makes use of Cas12a to precisely release target DNA fragments, which in this case were 43 uncharacterized BGCs from *Streptomyces* and *Bacillus* species, and later assembles them into DNA receivers harboring *lox*P sites. This construct will recircularize inside an *E. coli* that already has a helper plasmid, resulting in a BCG-containing plasmid that can be introduced to a host bacterium. This platform was shown to be efficient and robust, as all these BGCs were heterologously expressed in *B. subtilis*, *Streptomyces avermitilis*, or *Streptomyces lividans*, and 15 new NPs were identified. Six of those compounds were heterodimeric aromatic polyketides with a 5/6/6/6/5 pentacyclic carbon ring [104] and were named bipentaromycins. Four of these bipentaromycins were found to present antimicrobial activity against both Gram-negative and Gram-positive bacteria, including pathogens such as *E. faecium*, *S. aureus*, and *Bacillus anthracis*.

Another study was carried out by Yu et al. [92] on *Lysobacter enzymogenes* OH11. The importance of this microorganism relies on the fact that it is able to synthesize WAP-8294A. WAP-8294A is a family of 19 cyclic lipodepsipeptides with a previously known strong antibiotic activity against MRSA. The main disadvantage is that *L. enzymogenes* OH11 produces these compounds in low quantities and only under specific laboratory conditions, so the aim of this work was to obtain a strain capable of constitutively synthesizing them in higher concentrations. Unlike the other studies mentioned in this review, the modification did not involve deleting or editing a genetic element related to antibiotic production, but instead relied on trans-activating its expression. To achieve this, they used a modified Cas9, called dead-Cas9 or dCas9, which can bind to DNA but not cleave it. This protein was obtained by mutating the catalytic zone of the enzyme and fusing it with the RNA polymerase ω-subunit to target the promoter [105,106]. Using this CRISPR/dCas9ω3 system, five genes (*orf1-5*) related with WAP-8294A molecules were overexpressed. Additionally, five protective genes of *L. enzymogenes* were refactored into an operon under a strong promoter, in order to prevent the increased production of WAP-8294A from negatively affecting the bacteria [107]. As a result, the yields of three main compounds of the WAP-8294A family, WAP-8294A2, WAP-8294A1, and WAP-8294A4, increased 4, 6, and 9 folds, respectively. Among them, WAP-8294A2 (called lotilibcin) stands out for having reached clinical studies, in which it was tested against a wide group of multiresistant pathogens such as *S. aureus*, *S. epidermidis*, *S. pyogenes*, *S. agalactiae*, *S. pneumoniae*, *E. faecalis*, *Bacillus cereus*, and *Listeria monocytogenes*. Lotilibcin presented the best antibacterial activity against methicillin- and daptomycin-resistant *S. aureus*, with an MIC between 0.2 and 0.8 μg/mL.

The following study is centered around the production of microbial compounds, rather than antibiotic discovery and development. Pantoja Angles et al. [96] developed a CRISPR/Cas12 system that can shred the bacterial chromosome of E. coli, preventing it from replicating further but still allowing its metabolism to continue, what they call a chassis. With the addition of cargo genes of interest, the bacteria can be programmed to produce a specific compound, as shown by the production of the anticancer and antimicrobial drug violacein tested by the researchers. This shows that CRISPR/Cas systems can be used not only to perform specific genetic modifications, but also to allow the biomanufacturing of antibiotics, as well as other products.

### 3.2. Research on Fungi

Not all of the research is performed on bacteria, as there are a couple of studies that report genetic modifications on fungi using CRISPR/Cas, and the results of both involve fungal growth and the production of specialized metabolites, specifically two β-lactam antibiotics.

In the first study, Xu et al. [93] used *Acremonium chrysogenum* and analyzed the effect of growth regulator *acaxl2* on cephalosporin-C production. The arthrospore stage of this species has an active cell metabolism, and given that the strain *A. chrysogenum* FC^3^-5-23 is used to produce cephalosporin-C, understanding its growth, differentiation, and metabolite production is of industrial relevance. The regulator *acaxl2* is involved in the budding of fungal cells, and its deletion had been previously shown to stimulate arthrospore formation [108]. By knocking-out the *acaxl2* gene using a CRISPR/Cas9 system with homology-directed repair, they confirmed that the fungi grew in the shape of arthrospores and found that it had increased cephalosporin-C production. Interestingly, the same modification in a wild strain did not affect the production of this antibiotic. This can be explained by the different responses in the expression of the regulatory genes *acfkh1* and *cpcr1*, involved in both morphology and metabolism, as it was increased for both genes in the industrial strain’s mutant but not in the wild strain’s mutant. This shows not only the complexity of secondary metabolite production in fungi but also how different strains can have different regulatory systems.

In the second study, Gil-Durán et al. [94] studied the role of *pczl1* in *Penicillium rubens*, an industrial producer of penicillin. This gene codifies for a zinc finger protein with a Zn(II)_2_Cys_6_ domain—a type of regulatory protein very abundant in fungi—that regulates growth, apical germination, and specialized metabolite production in *Penicillium roqueforti* but had not been studied in *P. rubens*. Using a CRISPR/Cas9 system, previously developed for genetic modifications in fungi, to disrupt the *pczl1* gene in the industrial strain *P. rubens* Wis 54-1255, they determined that it is important for the organism’s growth and that it negatively regulates conidial germination. Moreover, they found that this protein is a positive regulator of penicillin’s biosynthetic pathway.

These works demonstrate that genetic regulation in fungi is complex and have also shown that CRISPR technologies are a useful tool to study fungal regulatory genes. Moreover, a relationship can be established between fungal growth and antibiotic production, even though its specific mechanisms are still an unknown matter that require further research.

## 4. Conclusions

CRISPR/Cas systems have been a very interesting research topic for years, and their development as genetic engineering tools has made way for a useful instrument that can be applied in virtually all fields, from healthcare (oncology, virology, AMR) [109,110,111] to food production and safety [112,113,114], as well as environmental detection of contaminants and pathogens [115,116,117]. This is because it is a genetic tool with a very high specificity in the target genetic region, and it can be very easily designed. Due to these advantages, it has been used to address the problem of antibiotic-resistant microorganisms, a worldwide problem that causes countless deaths every year [5]. In this review, we focused on the use of CRISPR/Cas against these pathogens in two different, but complementary, ways: (i) its use for the modification of resistant bacteria, either to sensitize them or to directly lyse them, or (ii) to obtain new antibiotics that replace those already existing and increase antibiotic production.

Firstly, we explained the techniques that can be used to modify pathogens. Despite the advantages of CRISPR/Cas, the constructions need to be introduced inside the microorganisms to be able to effectively carry out their action. As this step may limit the procedure, strategies involving phages and nanoparticles that serve as carriers have been explained. These delivery methods could facilitate the entry of the CRISPR/Cas system into the cell and its subsequent genetic alteration [48,118].

The second matter mentioned above is the procurement of higher yields of or new antimicrobial products using CRISPR/Cas to modify different species, highlighting actinomycetes. Actinomycetes, including those extensively investigated, can still be used as a source of new antibiotics or other useful bioactive products such as anticancer or immunosuppressant drugs [119], since more than 95% of their gene clusters coding for NPs are yet to be characterized [120]. Research on bacterial NPs has barely scratched the surface and still has a long way to go, and metagenomic, transcriptomic, metabolomic, and genome-mining approaches will help in that regard. In this sense, it is interesting to note that some studies were not strictly limited to the subject of antibiotic production or resistance, but also included approaches to gain insights into the complexity of the existing links between the regulation of metabolism and the regulation of the biosynthetic pathways [121,122]. This shows that CRISPR/Cas technologies can be used in all the steps regarding antibiotic development and production, from understanding genetic regulation and metabolism to improving industrial strains [123]. Most of the literature shown in this review is centered on bacteria, but fungi have also been of interest, resulting in extensive literature on the use of CRISPR/Cas technologies on fungi outside the topic of antibiotics [124,125,126]. There is diversity in the filamentous fungi for which CRISPR/Cas systems are designed, and the goals range from studying fungal genetic regulation to discovering and producing NPs, showing that CRISPR/Cas technologies can be successfully applied when it comes to fungi and antibiotics.

Out of the different CRISPR/Cas systems available, Cas9 is the system of choice in most studies, but a few make use of Cas12. Each has its own set of features, and one of the most relevant differences between them is that each one can recognize different targets, so having both at one’s disposal allows for higher precision of the cleavage and the subsequent modification [127]. Even though only Cas9 and Cas12 have been used on the topic of antibiotics, there are various Cas proteins on hand. One method based on CRISPR/Cas technology designed to avoid unwanted modifications is using nickase-based CRISPR precision editing. It is an alternative to the classic CRISPR/Cas, as it relies on a Cas9 nickase instead of a Cas9 nuclease, so no double-strand breaks are produced in the DNA. This prevents problems like deletions as a consequence of recombinations [128]. However, the efficiency of this technique was lower at first, which is why new strategies derived from the Cas9 nickase have recently been developed to improve its performance and to obtain techniques that allow gene screening, which can be used to discover new BGCs [129,130]. This CRISPR/Cas nickase system has been successfully used in *Streptomyces* [131], so it is an additional genetic engineering tool that can be applied to antibiotic discovery, and potentially AMR management too.

Nevertheless, in spite of their versatility, CRISPR/Cas systems still have their limitations, including off-target activity, and the delivery of these systems into the cell [20,42]. For this reason, CRISPR/Cas technology is still evolving, and there are multiple approaches to improve and optimize it. Some of them involve derived or alternative uses for Cas proteins, like CRISPR-guided transposons [42], or the previously mentioned use of inactivated Cas proteins as transcriptional regulators.

Newly developed machine learning and artificial intelligence (AI) tools have already proven to be beneficial too, whether by identifying possible off-target activity, improving efficiency, aiding in protein structure predictions, or modeling complex biological systems [132,133]. Although their main use is in health applications in cancer or genome editing, they can also be applied for the treatment of multidrug-resistant bacteria, by designing modified bacteriophages or sgRNAs more easily, among other products [134,135]. Therefore, the combination of these two technologies, with the ease of use and quick action of CRISPR/Cas and the efficient and direct design by AI, would help improve human health. However, ethical aspects must be taken into account when using this technology, for example, to modify plants or animals meant for human consumption in order to make them resistant to AMR bacteria [136,137]. In summary, it is not too difficult to predict that both CRISPR/Cas approaches will be very useful tools to effectively treat antibiotic-resistant pathogens in the near future.

## Figures and Tables

**Table 1:** Summary of the articles discussed in this review regarding the use of CRISPR/Cas in antibiotic development and production.

Host Microorganism	CRISPR Method	Modifications	Antibiotic	Result	Reference
*Streptomyces roseosporus* NRRL 15 998	CRISPR/Cas9	Activation of a BGC	Auroramycin	Discovery of a BGC	[85]
*Streptomyces* sp. SD85	CRISPR/Cas9	sceN knock-out	Sceliphrolactam	Verification that the BGC is involved in the formation of the antibiotic	[86]
*Streptomyces antibioticus* ZS	CRISPR/Cas9	Overexpression of regulator actO	Actinomycins D and V	Increase in the production of actinomycin D (4.4 fold) and actinomycin V (2.6 fold)	[87]
*Streptomyces rimosus* M4018	CRISPR/Cas9	Disruption of the zwf2 and devB genes	Oxytetracycline	Increase in antibiotic production of 36.8%	[88]
*Saccharopolyspora erythraea* NRRL 23338	CRISPR/Cas9	Replacement of promoters in ery gene cluster	Erythromycin	Increase in antibiotic production of 2.8 to 6 fold	[89]
*Amycolatopsis orientalis* AO-2	CRISPR/Cas12a	Substitution of a BGC	A82846B, a precursor to oritavancin	Increase in the production of the precursor	[90]
*Bacillus subtilis* 168	CRISPR/Cas9	Promoter substitution	Fengycin	Increased production of fengycin by 5.22%	[91]
*Lysobacter enzymogenes* OH11	CRISPR/dCas9-ω3	Overexpression of a BSC	Cyclic lipodepsipeptides WAP-8294A	Increased production of antibiotic up to 9 fold	[92]
*Acremonium chrysogenum* CGMCC	CRISPR/Cas9	Knock-out of a septum formation regulator, Acaxl2	Cephalosporin C	Increased production of cephalosporin C	[93]
*Penicillium rubens*	CRISPR/Cas9	Disruption of the pcz1 gene	Penicillin	Decrease in penicillin production	[94]
*B. subtilis* JH642 + sfp, *Streptomyces avermitilis* SUKA17, *Streptomyces lividans* TK24	CRISPR/Cas12a	Heterologous expression of BGCs	Bipentaromycins	Discovery of BGCs	[95]
*E. coli* BL21	CRISPR/Cas12	Shredding of the chromosome	Violacein	Establishment of a chassis for biomanufacturing	[96]
